# Impaired calcium mobilization in vascular smooth muscle of rats in septic shock

**DOI:** 10.1186/cc12978

**Published:** 2013-11-05

**Authors:** Sandra Crestani, Jose Eduardo da Silva Santos, Jamil Assreuy

**Affiliations:** 1 Department of Pharmacology, UFSC, Florianópolis, SC, Brazil

## Background

Calcium activity is essential to vascular smooth muscle contraction. Although it is well established that arteries from rats in septic shock present hyporesponsiveness to vasoconstrictor drugs, the role of calcium mobilization in this contractile dysfunction is far less investigated. We hypothesized that during septic shock calcium dynamics is changed and may have a role in the vascular dysfunction in sepsis.

## Materials and methods

Female Wistar rats (3 months old) were anesthetized by oxygen-isoflurane (3%) inhalation and subjected to cecal ligature and puncture surgery (CLP). Immediately after and every 12 hours rats received physiological saline solution (PBS 30 ml/kg, subcutaneously) and tramadol (5 mg/kg, subcutaneously). After 6 hours (CLP-6) or 24 hours (CLP-24) rats were killed, the aorta was harvested and cut in rings, the endothelium was removed and rings were mounted in baths. Rings were exposed to KCl 120 mM and phenylephrine (PE 1 µM). Aorta rings were kept in a modified depolarizing Krebs solution nominally Ca^2+ ^free and contracted by CaCl_2 _(1 to 100 mM). The same protocol were repeated in presence of thapsigargin (3 µM), DTNB (100 µM) or PTIO (100 µM). Different vessels were exposed to single concentrations of PE (1 μM) or caffeine (20 mM) in Ca^2+^-free solution, in the presence or absence of thapsigargin.

## Results

Maximal contraction (E_max_) induced by KCl or PE was reduced, especially in the CLP-24 group. Similarly, CaCl_2_-induced contraction was reduced (60%) in the CLP-24 group. Thapsigargin (sarcoplasmatic calcium reuptake blocker) and DTNB (sulphydryl oxidation) restored the contraction elicited by CaCl_2 _in septic rings, but without effect in control rings. PE-induced contraction in calcium-free solution was significantly reduced in CLP-24 rings (E_max _1.6 ± 0.4 g control vs. 0.3 ± 0.1 g CLP-24 rings). Thapsigargin did not change the hyporesponsiveness to PE but PTIO (nitric oxide scavenger) restored it partially. Caffeine-induced contraction in Ca^2+^-free solution was reduced in CLP-24 rings (0.2 ± 0.06 g control vs. 0.03 ± 0.01 g in CLP-24). Thapsigargin or PTIO restored the contraction induced by caffeine.

## Conclusions

These data suggest that in septic shock septic calcium mobilization is strongly impaired. Although preliminary, our results suggest that calcium channel nitrosylation and calcium reuptake may be reasons for the vascular hyporesponsiveness of septic shock.

